# Identification of Six Flavonoids as Novel Cellular Antioxidants and Their Structure-Activity Relationship

**DOI:** 10.1155/2020/4150897

**Published:** 2020-09-19

**Authors:** Qiang Zhang, Wenbo Yang, Jiechao Liu, Hui Liu, Zhenzhen Lv, Chunling Zhang, Dalei Chen, Zhonggao Jiao

**Affiliations:** Zhengzhou Fruit Research Institute, Chinese Academy of Agricultural Sciences, Zhengzhou, 450009 Henan, China

## Abstract

This study is aimed at determining the relationship of flavonoid structures to their chemical and intracellular antioxidant activities. The antioxidant activities of 60 flavonoids were investigated by three different antioxidant assays, including 2,2-diphenyl-1-picrylhydrazyl (DPPH) radical scavenging activity, oxygen radical absorption capacity (ORAC), and cellular antioxidant activity (CAA) assays. The result showed 6 flavonoids as good cellular antioxidants evaluated for the first time. The cellular antioxidant activities of compounds 7-methoxy-quercetin, 3-*O*-methylquercetin, 8-hydroxy-kaempferol, quercetin-3-*O*-*α*-arabinofuranose, kaempferol-7-*O*-glucopyranoside, and luteolin6-*C*-glucoside were linked with the upregulation of antioxidant enzyme activities (superoxide dismutase, catalase, and glutathione peroxidase). A structure-activity relationship suggested that 2,3-double bond, 4-keto groups, 3′,4′-catechol structure, and 3-hydroxyl in the flavonoid skeleton played important roles in the antioxidant behavior. Furthermore, the cell proliferative assay revealed a low cytotoxicity for 3-*O*-methylquercetin. The present results provide valuable information for the dietary application of flavonoids with different structures for high antioxidant.

## 1. Introduction

The reactive oxygen species (ROS) are known to damage the tissues of the body, which leads to disturb the established order on the body system. The ROS attacked biomolecules like DNA, lipids, and proteins to free radical damage, which stimulated the development of many diseases, such senility, angiocardiopathy, and cancer [1]. At present, researchers have found that flavonoid consumption can improve cancer and cardiovascular diseases [2]. There are inverse relationship between dietary flavonoids and chronic diseases, which displayed the importance of studying flavonoids [3].

Flavonoids is one of the most abundant phenolic compounds in various fruits, vegetables, grains, spices, beverages, and medicinal plants, which are structured by a C_6_-C_3_-C_6_ skeleton labeled with the rings A, B, and C (Table 1). The subclasses included flavones, flavonols, flavanones, flavanols, anthocyanidins, and isoflavonoids [4]. Many researchers have discovered a wide range of biological activities of the flavonoids in prevention and relieve various diseases such as obesity, diabetes, cancer, angiocardiopathy, and heart diseases [5–7]. Therefore, the flavonoids were considered to be candidates for these disease management due to the ROS and iNOS caused [8]. The capacity of flavonoids depends on their substituent groups, the number of hydroxyl groups, other substitutions, and conjugations. In addition, quercetin, kaempferol, rutin, hesperidin, naringin, genistein, phloretin, isoquercitrin, taxifolin, epicatechin, cyanidin chloride, and their derivatives were widely distributed in apples, blueberries, cherries, grapes, tea, citrus, peppers, red wine, chocolate, etc., which has extensive biological activity [9–11]. However, to our knowledge, systematic studies on differences in the antioxidant ability of various flavonoids and the structure-activity relationships are still scarce. In particular, the influence between different structural flavonoids and the antioxidant enzyme activities (superoxide dismutase (SOD), catalase (CAT), and glutathione peroxidase (GSH-Px)) has rarely been studied.

Therefore, we have chosen 60 flavonoids, which have the diversity of their core structures and substitution patterns, which contribute to systematic studies on the differences in chemical and cell-based antioxidant assays in this work. The antioxidant activities of a series of flavonoids (Table 1) which are commonly found in diet, including flavones, flavonols, flavanones, flavanols, flavanes, chalcones, and anthocyanidins, were examined by 2,2-diphenyl-1-picrylhydrazyl radical scavenging activity, oxygen radical absorption capacity, and cellular antioxidant activity assays. The structure-activity relationship of different structures of dietary flavonoids was analyzed for obtaining the substructures with high antioxidant activity. The cellular antioxidant activity assay was closer to physiological conditions for giving an extensive evaluation of the antioxidant. Moreover, the cytotoxicity and antiproliferative activity assays were also measured. This study has provided the theoretical foundation for the structural modification of flavonoids as effective antioxidant.

## 2. Material and Methods

### 2.1. Chemical and Reagents

Dimethyl sulfoxide (DMSO), 2,2-diphenyl-1-picrylhydrazyl (DPPH), Trolox, fluorescein sodium salt, 2′,7′-dichlorfluorescin diacetate (DCFH–DA), and 2,2-azobis (2-amidinopropane) dihydrochloride solution (ABAP) were purchased from Sigma Chemical Co. (Sigma-Aldrich, St. Louis, MO, USA). Flavonoid standards were purchased from Solarbio Science & Technology Co., Ltd. (Beijing, China). Phosphate buffer (PBS), MEM/EBSS, foetal bovine serum (FBS), penicillin, and streptomycin were purchased from HyClone (Logan, UT, USA). Cell Counting Kit-8 was obtained from Dojindo China Co., Ltd. (Shanghai, China). Kits for the determination of superoxide dismutase (SOD), glutathione peroxidase (GSH-Px), and catalase (CAT) were purchased from Beyotime Biotechnology (Shanghai, China).

### 2.2. Oxygen Radical Antioxidant Capacity (ORAC) Assay

The ORAC assay was evaluated as previously described by Cao et al. with some modifications [12, 13]. 50 *μ*L of samples or Trolox with different concentrations and the fluorescein solution was added to a 96-well microplate, which was incubated at 37°C for 10 min. Then, 50 *μ*L of 119 mM AAPH (freshly prepared) was added to each well. The fluorescence generation was measured using a microplate reader at excitation of 485 nm and emission of 520 nm for 60 cycles every 2 min. The ORAC values were calculated by the regression equation between the Trolox concentration and the net area under the curve (expressed as *μ*mol Trolox eq/*μ*mol sample).

### 2.3. DPPH Radical Scavenging Activity

This assay was conducted as previously described by Wen et al. with some modifications [14]. DPPH was freshly prepared in methanol at a concentration of 0.1 mM. The solution (20 *μ*L) containing the tested compounds with different concentrations was added into the DPPH solution (180 *μ*L) in the 96-well plates. The plates were incubated at 37°C for 30 min in the dark, and the absorbance value was recorded at 515 nm. The IC_50_ value was calculated on the scavenging activity against DPPH radical.

### 2.4. Cellular Antioxidant Activity

#### 2.4.1. Determination of Cellular Antioxidant Activity (CAA)

The CAA assay was tested as described previously [15]. 6 × 10^4^ cells/well of HepG2 cells were seeded at a 96-well microplate with 100 *μ*L of growth medium/well. The cells were primarily treated with 100 *μ*L of medium containing the tested compounds and DCFH-DA (25 *μ*M) for 1 h at 37°C. Then, the cells were washed with PBS and treated with 100 *μ*L of 600 *μ*M ABAP (dissolved in HBSS), and the 96-well microplate was immediately placed into an Infinite SpectraMax i3x Multi-Mode Detection plate-reader at 37°C. The fluorescence reading was measured at an emission of 535 nm and excitation of 485 nm every 5 min for 1 h. Quercetin was used as positive control; the EC_50_ values were expressed in micromoles of quercetin equivalents per 100 *μ*mol of tested compounds (*μ*mol QE/100 *μ*mol of sample).

#### 2.4.2. Activity Determinations of Cellular Antioxidant Enzymes

HepG2 cells were seeded (1 × 10^6^ cells/well) in six-well plates. After incubation for 24 h, the cells were pretreated with different concentration samples. Medium was washed by PBS and treated with 600 *μ*M ABAP. The cells were collected and treated with cell lysis buffer (20 mM Tris at pH 7.5, 150 mM NaCl and 1% Triton X-100) at 4°C. The lysed cells were used to measure the intracellular activities of SOD, CAT, and GSH-Px by kits according to the manufacturer instructions (Wen 2015). Cells without sample and ABAP treatment were used as positive control (PC), while cells treated with ABAP but not sample were used as negative control (NC).

#### 2.4.3. Cytotoxicity and Antiproliferative Activity Assays

The cytotoxicity and antiproliferative activity assays were performed by using the CCK-8 assay kit [16]. Briefly, HepG2 cells were cultured at a density of 4 × 10^4^ cells/well or 2.5 × 10^4^ cells/well in a 96-well microplate with growth medium. After incubation at 37°C, the growth medium is treated with 100 *μ*L of growth medium containing different concentrations of tested compounds for 24 h or 72 h. The wells having growth medium without the tested compound served as control. Then, the cells were incubated with 10 *μ*L/well CCK-8 solutions for 2 h at 37°C. The absorbance values of each well were measured at 450 nm using a microplate reader (SpectraMax i3x, ForteBio Analytics Co., Ltd., USA). The cytotoxic activity and antiproliferative effects of the tested compound was calculated as

(1) $$\begin{array}{c} \text{Cytotoxicity }\left(\%\right)=\left(1-\text{As}/\text{Ac}\right)\times 100\%, \end{array}$$

(2) $$\begin{array}{c} \text{Cell proliferation }\left(\%\right)=\left(\text{As}/\text{Ac}\right)\times 100\%, \end{array}$$

where As is the absorbance of the well with compound; Ac is the absorbance of control.

### 2.5. Statistical Analysis

All data were presented as mean ± standard deviation for triplicate analyses (*n* = 3). One-way analysis of variance (ANOVA) was used to compare the means. Differences were considered significant at *P* < 0.05. All statistical analysis was performed using IBM SPSS statistical software 21.0 (IBM Corporation, NY, USA).

## 3. Results and Discussion

### 3.1. Antioxidant Capacity

#### 3.1.1. Chemical Antioxidant Activity

**Figure 1:**
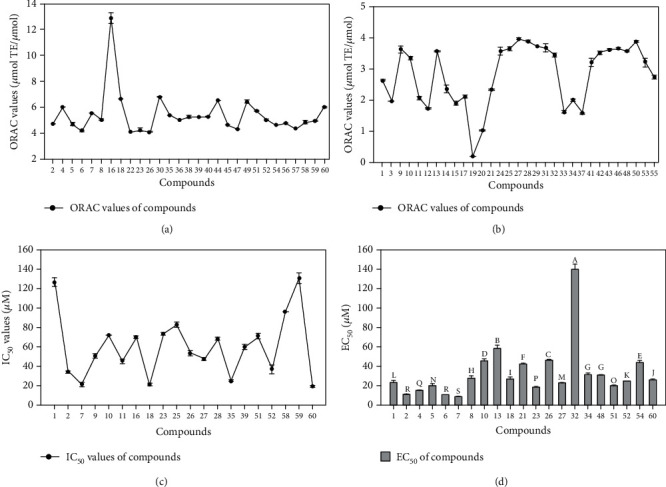
The antioxidant activities of flavonoids determined by ORAC (a, b), DPPH (c), and the cellular antioxidant (d) assays. The IC_50_ and EC_50_ of compounds that were not in the Figure were >200 *μ*M. The data are presented as the mean with standard deviation (SD) bar of three replicates. The values having no letters in common are significantly different (*P* < 0.05). The data was listed in Table S1.

The antioxidant activities of flavonoids were assessed by ORAC and DPPH assays. Quercetin, a well-known antioxidant, was used as positive control. The ORAC assay is based on the oxidation of a fluorescent probe (fluorescein) by radicals coming from the spontaneous decomposition of AAPH. The ORAC process is a classical oxidation process for hydrogen atom transfer [17]. As shown in Figure 1(a), strong oxygen radical absorbance capabilities were observed in compounds 2, 4-8, 16, 18, 22-23, 26, 30, 35-36, 38-40, 44-45, 47, 49, 51-52, 54, and 57-60, with their ORAC values ranging from 4.07 to 12.85 *μ*mol TE/*μ*mol. Among the compounds, compound 16 (12.85 ± 0.42 *μ*mol TE/*μ*mol) was found to possess the highest peroxyl radical scavenging activity, followed by compounds 30, 18, 44, 49, and 60 (6.80 ± 0.42, 6.64 ± 0.03, 6.52 ± 0.15, 6.43 ± 0.14, 6.02 ± 0.14 *μ*mol TE/*μ*mol, respectively). Compounds 2, 4-8, 22-23, 26, 35-36, 38-40, 45, 47, 51, 52, 54, and 56-59 were not significantly different from compound 60. The ORAC values of compounds 1, 3, 9-15, 17, 19-21, 24-25, 27-29, 31-34, 37, 41-43, 46, 48, 50, 53, and 55 ranged from 0.21 to 3.97 *μ*mol TE/*μ*mol (Figure 1(b)). However, compound 19 (0.21 ± 0.01 *μ*mol TE/*μ*mol) had the lowest antioxidant activities in the ORAC assay.

DPPH assay is based on the reduction of DPPH^•^ in the presence of a hydrogen-donating antioxidant, leading to form DPPHH. The DPPH radical scavenging activities of tested flavonoids are shown in Figure 1(c). Compounds 2, 7, 9-11, 16, 18, 23, 25-28, 35, 39, 51-52, 58, and 60 exhibited a strong DPPH radical scavenging activity with their IC_50_ value ranging from 19.13 to 96.03 *μ*M, while compounds 1 and 59 (126.48 ± 4.26, 129.99 ± 5.55 *μ*M, respectively) had a much lower radical scavenging activity. The others had no antioxidant activity. Among the tested flavonoids, compounds 2, 7, 18, 35, 52, and 60 were found to possess the highest DPPH radical scavenging activity (34.03 ± 0.61, 21.52 ± 1.90, 21.26 ± 1.33, 25.25 ± 0.62, 36.83 ± 4.26, 19.13 ± 0.62 *μ*M, respectively), followed by compounds 9-11, 16, 23, 25-28, 39, 51, and 58, which were 50.87 ± 2.14, 71.68 ± 0.06, 45.07 ± 2.12, 69.97 ± 1.44, 73.23 ± 0.75, 82.41 ± 2.88, 53.34 ± 2.64, 47.68 ± 1.60, 68.26 ± 1.37, 59.55 ± 3.12, 70.80 ± 2.31, 96.03 ± 0.13 *μ*M, respectively.

#### 3.1.2. Cellular Antioxidant Activity

**Figure 2:**
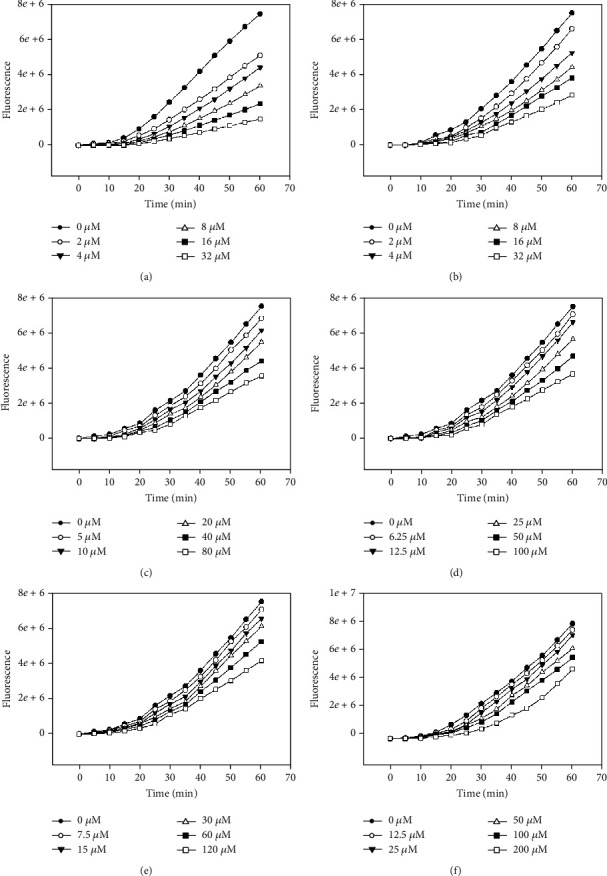
Peroxyl radical-induced oxidation of DCFH to DCF in HepG2 cells and the inhibition of oxidation by compounds 2 (a), 5 (b), 8 (c), 10 (d), 13 (e), and 32 (f) over time, using the protocol having no PBS wash.

Chemical antioxidant assays are difficult to exactly reflect the antioxidant activity *in vivo*. Comparatively, the advantage of CAA assay was to simulate cellular biological processes which include uptake, distribution, and metabolism. CAA assay was conducted to quantify the capacity of the analyte to prevent the formation of DCF by AAPH-induced peroxyl free radical in HepG2 cells. The level of cellular fluorescence in CAA assay was relevant to the degree of the DCFH oxidation, which demonstrated that a decrease in fluorescence caused by the analyte shows a cellular antioxidant capacity [18]. The cellular antioxidant activities of compounds 2, 5, 8, 10, 13, and 32 were identified for the first time in this work. The kinetics of DCFH oxidation in HepG2 cells induced by peroxyl radicals are displayed in Figure 2. The results illustrated that the increase in fluorescence due to DCF formation was inhibited by tested flavonoids in a dose-dependent manner.

The EC_50_ of the compounds are listed in Figure 1(d). In this study, the antioxidant activity of compound 2 was as good as the positive reference, quercetin, which EC_50_ was 9.84 ± 0.34 *μ*M. The EC_50_ values of compound 5, 8, 10, 13, and 32 were 19.53 ± 1.48, 27.12 ± 2.47, 45.12 ± 2.12, 57.78 ± 3.12, and 139.21 ± 5.21 *μ*M, respectively.

Compound 2 showed an unexpected effect on the inhibition of DCF formation. Compounds 1 2, 4, 5, 6, 8, 10, 13, 21, 23, 26, 27, 32, and 34 have a similar structure to quercetin had and no hydroxyls exist on C-3, C-3′, and C-5′. The structure difference led to apparent changes in the cellular antioxidant assay. Quercetin (7) and compound 2 had a strong cellular antioxidant activity. The loss of C-3′ or C-5′ hydroxyls influenced the cellular antioxidant activity [19]. The loss of C-3′ or C-5′ hydroxyls destroys the *ortho*-dihydroxyl structure and thereby decreases the antioxidant activity because *ortho*-dihydroxyl contributes much to the radical scavenging effect of flavonoid [20]. Therefore, compound 1 [21], compound 4 [22], compound 6 [18], compounds 8, 13, 21 [23], and 34 [24] showed a significant difference from compounds 2 and 7. The loss of 3-hydroxyl moiety also decreased the cellular antioxidant activity, as indicated by compounds 5, 10, 21 [23], 23 [18], compounds 26 [25] and 32. Compounds 48 [18], 51, 52 [26], and 60 [18] had strong activities on account of the number of hydroxyl group. Moreover, an additional 5′-hydroxyl group in the B-ring, as seen compound 18 [18], has been revealed to decrease antioxidant activity. The presence of *O*-glycoside decreased the antioxidant activity, as indicated by compounds 7 and 27 [25].

A significant cellular antioxidant effect was observed for compounds 2, 5, 8, 10, and 13 which showed a consistent dose-dependent antioxidant effect. Unlike other methods commonly used for measuring chemical antioxidant activity, this assay has been developed a more biologically representative protocol. Antioxidants can act at the cell membrane to break peroxyl radical chain reactions at the cell surface or can be uptaken by the cell and react with ROS intracellularly [19]. The efficiencies of membrane binding and cell uptake are two important factors influencing the antioxidant activity of the tested chemical.

It is noteworthy that although the CAA assay represents a reliable and cost-effective approach to evaluate the potential biological activity of dietary flavonoids on cellular level and conveys important reference value to the functional food development, it does not fully reflect the in *vivo* metabolism of these compounds. The metabolic process of food-derived polyphenols in the human body could be complicated because they might be extensively degraded and metabolized by various gut enzymes and microflora. The resulting metabolic products of dietary flavonoids would also contribute to biological activities once they are released into the systemic circulation [27].

### 3.2. Structure-Antioxidant Activity Relationship

#### 3.2.1. Hydroxyl Groups

The spatial arrangement of substituents is more important than the flavan backbone alone in the antioxidant activity. Consistent with most polyphenolic antioxidants, both the number and positioning of the B-ring hydroxyl groups in flavonoids substantially influence the mechanisms of antioxidant activity. Especially, a 3′,4′-catechol structure in the B-ring strongly enhances the antioxidant activity [28]. In the CAA assay, compound 7 (quercetin), which has a 3′,4′-*O*-dihydroxyl group, had the highest activity with an EC_50_ of 8.77 ± 0.09 *μ*M. Compounds 2, 5, and 23 had the same skeleton with small moiety differences, which had only slightly lower activities than quercetin. Compound 60 had strong activities on account of the number of hydroxyl group. Compounds 4 and 40, which have two hydroxyl groups in the B-ring, had much lower activity (15.23 ± 0.32, >200 *μ*M) than quercetin. The presence of an *m*-diphenolic moiety reduced activity compared to the *ortho* configuration in the previous study [19]. The presence of the *ortho*-dihydroxyl group in the B-ring has stabilized the antioxidant performance owing to participating electron delocalization and hydrogen bonds between 3′- and 4′-hydroxyls [29]. Compared to quercetin, the 5′-hydroxyl group of compound 18 decreased the cellular antioxidant activity; however, the DPPH radical scavenging activity and ORAC activity were little changed. Compounds 58-59 had lower antioxidant activity than compound 60. In the DPPH and ORAC assays, compounds 2, 23, and 60 showed good activity, which owned hydroxyls but not be affected by other groups. The compounds 4, 5, 40, 58, and 59 have good activity in ORAC assay and lower DPPH radical scavenging activity, but compounds 4 and 5 gained good cellular antioxidant activity, which illustrated the other groups, membrane association, and uptake in cell also played important roles in different antioxidant assays. The presence of a galloyl group in the compound 60 imparted it with high activity in all assays. These results indicate that 3′,4′-*O*-dihydroxyl group is an important structure feature of substantial antioxidant activity for flavonoids in the CAA assay. This finding was in consistent with the results of DPPH and ORAC assay. Previous researches also suggested that a B-ring catechol group is essential for high antioxidant activity [30, 31].

#### 3.2.2. C/O-Glycoside and O-Methylation

Moreover, an additional *C*/*O*-glycoside or *O-*methylation, as seen in compounds 1, 3, 9-17, 19, 25-33, 36, 41-44, 48-49, and 56-57, has been revealed to decrease antioxidant activity on account of a prooxidant counteracting their antioxidant effect [32]. Compounds 9-17, 25-33, 41-44, 48-49, and 56-57 showed lower cellular antioxidant activity than their aglycones, which indicated the *C*/*O*-glycoside decreased the antioxidant activity [6]. This finding was in consistent with the results of DPPH and ORAC assay. Owing to the *O-*methylation group, compounds 1, 3, 19, and 36 had lower antioxidant activities in three assays. Compounds 9-11, 25, 28-33, 41-44, 48-49, and 56-57 have good ORAC activity and lower cellular antioxidant activity, which revealed the degree of membrane association and uptake in cell, owing to the structure of flavonoids, polarity, and solubility.

#### 3.2.3. The 2,3-Double Bond, 4-Keto Group, and 3-Hydroxyl Moiety

For flavonoids with a B-ring catechol group, the loss of any of the C-ring functional group, the 2,3-double bond, 4-keto group, or 3-hydroxyl moiety lead to decrease antioxidant activity [14]. In the CAA assay, the antioxidant activity of compounds 5, 9-10, 16, 23-26, 28-29, 31-32, 38-40, and 53-54 with 2,3-double bond and 4-keto groups decreased due to the loss of 3-hydroxyl moiety. This finding was in consistent with the results of the DPPH assay. However, the 2,3-double bond of C-ring did not influence the activity in the ORAC assay. Meanwhile, the 2,3-double bond of compounds 7, 23, and 39 would be further impacted than 3-hydroxyl moiety in the CAA assay. The big difference of flavonoids in ORAC, DPPH, and the cell assay suggested some compounds were not so effective in the model of CAA, and this different phenomenon provides information on the degree of membrane association and uptake in cell, owing to their structure, polarity, and solubility.

### 3.3. Effect on Intracellular Antioxidant Enzymes

**Figure 3:**
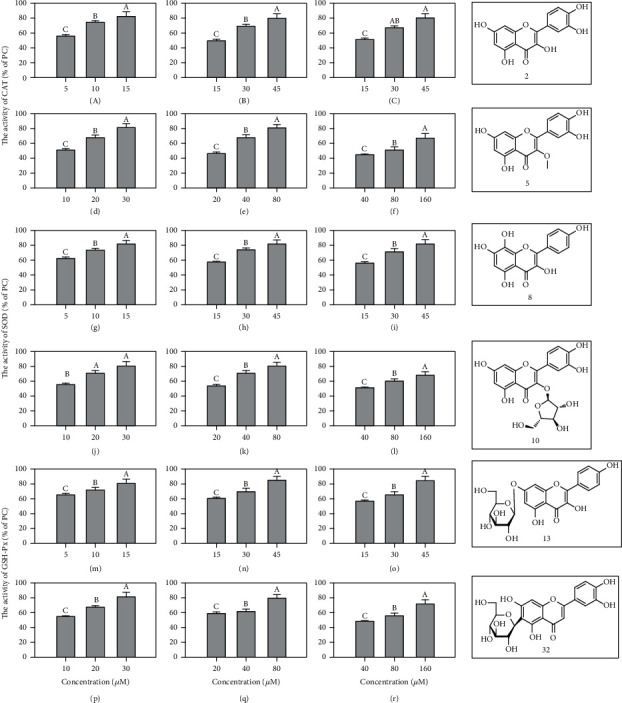
The rate and structures of the compounds 2 (a, g, p), 5 (b, h, q), 8 (c, i, r), 10 (d, j, s), 13 (e, k, t), and 32 (f, o, u) of PC value on the activities of antioxidant enzymes. The activities of CAT, SOD, and GSH-Px of the PC were 106.82 ± 5.32, 4.86 ± 0.84, and 33.3746 ± 2.25 U/mg protein, respectively. The activities of CAT, SOD, and GSH-Px of the NC were 45.11 ± 2.21, 2.12 ± 0.21, and 14.4231 ± 1.25 U/mg protein, respectively. The data are presented as the mean with standard deviation (SD) bar of three replicates. The values having no letters in common are significantly different (*P* < 0.05).

The overproduction of ROS caused the imbalance of the intracellular oxidation stress, which may result in damage to cell. It is a leading factor contributing to chronic diseases, which include aging, angiocardiopathy, hypertension, and neurodegenerative diseases [33]. ABAP-induced ROS generation can cause an imbalance of intracellular antioxidant defense system, and SOD, CAT, and GSH-Px were the major radical-scavenging enzymes. In order to further measure the intracellular antioxidant mechanisms of flavonoids, the activities of SOD, CAT, and GSH-Px were determined. Cells without sample and ABAP treatment were used as positive control (PC), while cells treated with ABAP but not sample were used as negative control (NC). The data are shown in Figure 3. The SOD, CAT, and GSH-Px activities of NC cells were 43.47 ± 3.12%, 42.24 ± 3.45%, and 43.21 ± 4.21% of the PC cells, respectively. This suggested that ABAP caused oxidative stress in HepG2 cells. However, pretreating cells with compounds 2, 5, 8, 10, 13, and 32 before ABAP treatment prevented the activity decrease of antioxidant enzyme activities. The cells pretreated with 5 *μ*M compound 2, 15 *μ*M compound 5 and 8, 10 *μ*M compound 10, 20 *μ*M compound 13, or 40 *μ*M compound 32 showed an insignificant increase in SOD activity, while a significant increase activity was found at a higher concentration compared to NC cells. Similarly, compounds 2, 5, 8, 10, 13, and 32 increased the CAT and GSH-Px activities in a dose-dependent manner. The CAT activities were 56.58 ± 3.25%, 74.98 ± 4.25%, and 83.10 ± 4.54%, and the GSH-Px activities were increased by 65.09 ± 3.21%, 71.88 ± 4.23%, and 81.24 ± 5.65% of PC value in cells pretreated with 5, 10, and 15 *μ*M compound 2. The CAT activities were 49.55 ± 3.21%, 69.34 ± 5.15%, and 80.67 ± 7.13%, and the GSH-Px activities were 59.74 ± 3.23%, 69.08 ± 4.27%, and 83.44 ± 4.18% of PC value in cells pretreated with 15, 30, and 45 *μ*M compound 5. The CAT and GSH-Px activities of compound 8 were 50.86 ± 2.23%, 67.01 ± 5.32%, and 80.86 ± 5.21%, 54.02 ± 3.02%, 63.46 ± 2.51%, and 82.02 ± 5.35% of PC value, respectively. Meanwhile, The CAT activities were 51.90 ± 3.21%, 68.95 ± 4.22%, and 81.82 ± 4.25%, and the GSH-Px activities were 54.37 ± 3.05%, 68.21 ± 4.25%, and 81.82 ± 5.52% of PC value in cells pretreated with 10, 20, and 30 *μ*M compound 10. The percentage value of compound 13 was similar to compound 10. However, The CAT and GSH-Px activities of compound 32 were 44.93 ± 2.23%, 52.68 ± 3.42%, and 68.35 ± 3.72%, 47.79 ± 3.28%, 55.99 ± 3.57%, and 71.20 ± 4.28% of PC value, respectively. The results were consistent with the CAA assay, and the compounds have the better cellular activity; the enzyme activities were higher. Therefore, the structure-activity relationship of intracellular antioxidant enzymes was the same as the CAA assay.

A previous study indicated that flavonoids can modulate intracellular antioxidant enzyme activities. Diosmetin is a bioflavonoid found in citrus fruits that has strong cellular antioxidant activity and can regulate the intracellular antioxidant enzyme activities to prevent the generation of intracellular ROS, thus effectively attenuate AAPH-induced oxidative stress in erythrocytes [34]. Butin was isolated from several medicinal herbs and reported to protect the cell against H_2_O_2_-induced DNA damage through restoring the activity and expressions of cellular antioxidant enzymes [35]. In this work, compounds 2, 5, 8, 10, 13, and 32 could significantly improve the activities of SOD, CAT, and GSH-Px. This could be one of the antioxidant mechanisms for compounds.

### 3.4. Cytotoxicity and Antiproliferative Activity

**Figure 4:**
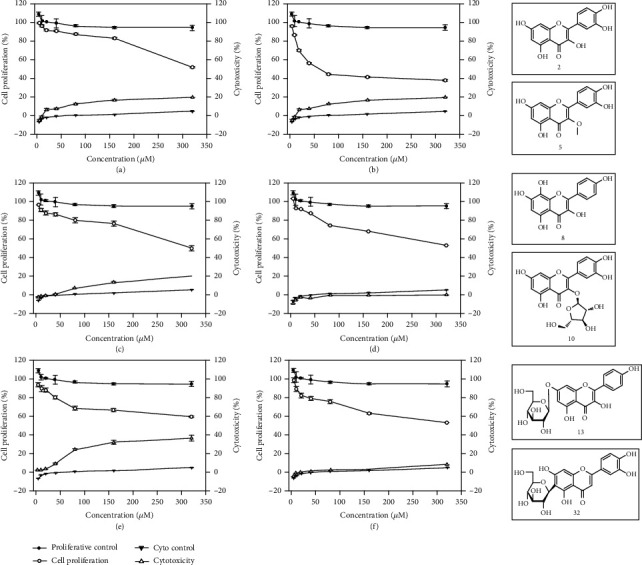
The antiproliferative activities, cytotoxicities, and structures of compounds 2 (a), 5 (b), 8 (c), 10 (d), 13 (e), and 32 (f) against HepG2 cells. The data are presented as the mean with standard deviation (SD) bar of three replicates. Bars with no letters in common were significantly different (*P* < 0.05).

The HepG2 cells were selected to determine the antiproliferative activities and cytotoxicities of compounds 2, 5, 8, 10, 13, and 32. As shown in Figure 4, compounds 2, 5, 8, 10, 13, and 32 had no significant effects in the range of 10-160 *μΜ*, while the compound 13 showed slight cytotoxicity at higher concentration. The results indicated that the reduced fluorescence in the CAA assay was not from cytotoxicity. The compound 5 showed potent antiproliferative activities against HepG2 cells. The IC_50_ values were 90.72 ± 2.45 *μΜ* to HepG2 cells, while others were more than 400 *μΜ*.

In the assay of cellular antioxidant, compound 5 has been recognized as a good antioxidant. Meanwhile, in the cancer cell proliferation assay, compound 5 could inhibit the proliferation of cancer cells. This result suggested that the 3-methoxyl group in the tested compounds play an important role in the antiproliferative activity compared to compounds 2 and 5 [36]. It could decrease the cellular antioxidant activity, but improve the antiproliferative activity against cancer cell. As confirmed by literature [37, 38], *O*-glycosidation usually decreases the antiproliferative activity of flavonoids compared to compounds 2, 10, 13, and 32. Compared to compounds 2, 5, and 8, the addition of the hydroxyl group at C-3, C-3′, and C-8 decreased the antiproliferative activity [39]. And the previous study reported that the C2-C3 double bond and the lack of C-6 hydroxyl group were the structural features needed for the antiproliferative activity of flavonoids [40]. However, the antiproliferative activity also has been influenced by other groups. Compound 5, as reported, could protected normal lung cells from H_2_O_2_-induced ROS formation, membrane damage, and DNA damage. Meanwhile, it also increased the expression of p-p38, Nrf2, and SOD [41]. All these results suggested a potential application of flavonoids in anticancer drugs and cosmetic products.

## 4. Conclusions

A series of flavonoids with different structures were used to determine their chemical and intracellular antioxidant activities, among which the cellular antioxidant activities of compounds 2, 5, 8, 10, 13, and 32 were identified and characterized for the first time in this work. Compounds 2 and 5 potent presented an unexpected cellular antioxidation behavior, which has an order of magnitude as the quercetin. Their intracellular antioxidant properties were related to the upregulation of endogenous antioxidant enzyme activities and inhibition of ROS generation. The 2,3-double bond, 4-keto groups, 3′,4′-catechol structure, and 3-hydroxyl in the flavonoid skeleton play important roles in the antioxidant behavior. Furthermore, the cell proliferative assay revealed a slightly cytotoxicity for compound 5. Therefore, compound 5 would be appropriate for the use of nutraceutical in the future.

## Figures and Tables

**Table 1:** The chemical structures of 60 flavonoids.

No	Flavonoids	Core structure	Substructure
	Flavone	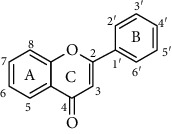	R=H
1	Isorhamnetin		R_3_, R_5_, R_7_, R_4__′_=OH, R_5__′_=OCH_3_
2	Rhamnetin		R_3_, R_5_, R_4__′_, R_5__′_=OH, R_7_=OCH_3_
3	Kaempferide		R_3_, R_5_, R_7_=OH, R_4__′_=OCH_3_
4	Morin		R_3_, R_5_, R_7_, R_4__′_, R_6__′_=OH
5	3-*O*-methylquercetin		R_3_= OCH_3,_ R_5_, R_7_, R_4__′_, R_6__′_=OH
6	Kaempferol		R_3_, R_5_, R_7_, R_4__′_=OH
7	Quercetin		R_3_, R_5_, R_7_, R_4__′_, R_5__′_=OH
8	Herbacetin		R_3_, R_5_, R_7_, R_8_, R_4__′_=OH
9	Myricitrin		R_3_=Orha, R_5_, R_7_, R_3__′_, R_4__′_, R_5__′_=OH
10	Avicularin		R_3_=Oara, R_5_, R_7_, R_3__′__,_ R_4__′_=OH
11	Trifolin		R_3_=Oglc, R_5_, R_7_, R_3__′_=OH
12	Kaempferol-4′-*O*-glucopyranoside		R_3_, R_5_, R_7_=OH, R_4__′_=Oglc
13	Kaempferol-7-*O*-glucopyranoside		R_3_, R_5_=OH, R_7_=Oglc, R_4__′_=OH
14	Kaempferol-3-*O*-arabinoside		R_3_=Oara, R_5_, R_7_, R_3__′_=OH
15	Isorhamnetin-3-*O*-glucopyranoside		R_3_=Oglc, R_5_, R_7_, R_3__′_=OH, R_4__′_=OCH_3_
16	Rutin		R_3_=Orha, R_5_, R_7_, R_4__′_, R_5__′_=OH
17	Spiraeoside		R_3_, R_5_, R_7_, R_5__′_=OH, R_4_=Oglc
18	Myricetin		R_3_, R_5_, R_7_, R_3__′_, R_4__′_, R_5__′_=OH
19	Tangeretin		R_5_, R_6_, R_7_, R_8_, R_4__′_=OCH_3_
20	Chrysin		R_5_, R_7_=OH
21	Baicalein		R_5_, R_6_, R_7_=OH
22	Apigenin		R_5_, R_7_, R_4__′_=OH
23	Luteolin		R_5_, R_7_, R_3__′_, R_4__′_=OH
24	Cynaroside		R_7_=Oglc, R_3__′_, R_4__′_=OH
25	Myricetin-3-*O*-galactoside		R_3_=Ogal, R_5_, R_7_, R_3__′_, R_4__′_, R_5__′_=OH
26	Quercetin-3-*O*-galactoside		R_3_=Ogal, R_5_, R_7_, R_3__′_, R_4__′′_=OH
27	Quercetin-3-*O*-rhamnoside		R_3_, R_5_=OH, R_7_=Orha, R_3__′_, R_4__′_=OH
28	Quercitrin		R_3_=Orha, R_5_, R_7_, R_3__′_, R_4__′_=OH
29	Isoquercitrin		R_3_=Oglc, R_5_, R_7_, R_3__′_, R_4__′_=OH
30	Vitexin		R_5_=Cglc, R_6_, R_8_, R_4__′_=OH
31	Orientin		R_8_=Cglc, R_5_, R_7_, R_3__′_, R_4__′_=OH
32	Isoorientin		R_4_=Cglc, R_5_, R_7_, R_3__′_, R_4__′_=OH
33	Isovitexin		R_5_, R_7_, R_4__′_=OH, R_6_=Cglc
34	Galangin		R_3_, R_5_, R_7_=OH
35	Fisetin		R_3_, R_7_, R_3__′_, R_4__′_=OH
36	Diosmetin		R_5_, R_7_, R_3__′_=OH, R_4__′_=OCH_3_
37	Genkwanin flavanones		R_5_, R_4__′_=OH, R_7_=OCH_3_ R=H

38	Dihydromyricetin	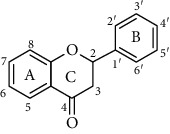	R_3_, R_5_, R_7_, R_3__′_, R_4__′_, R_5__′_=OH
39	Taxifolin		R_3_, R_5_, R_7_, R_4__′_, R_5__′_=OH
40	Dihydromorin		R_3_, R_5_, R_7_, R_4__′_=OH
41	Neohesperidin		R_5_, R_3__′_=OH, R_7_=Oglcgla, R_5__′_=OCH_3_
42	Narirutin		R_7_=Oglcgla, R_4__′_=OH
43	Hesperetin		R_5_, R_7_, R_4__′_=OH, R_5__′_=OCH_3_
44	Hesperidin		R_5_, R_5__′_=OH, R_7_=Oglcgla, R_4__′_=OCH_3_
45	Naringenin		R_5_, R_7_, R_4__′_=OH
46	Liquiritigenin		R_7_, R_4__′_=OH

	Chalcone	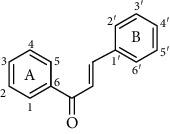	R=H
47	Neohesperidin dihydrochalcone		R_3_, R_3__′_, R_6__′_=OH, R_4__′_=Oglcgla
48	Phloretin		R_1,_ R_3_, R_5_, R_4__′_=OH
49	Phlorizin		R_1_, R_3_, R_4__′_=OH, R_5_=Oglc
50	Isoliquiritigenin		R_1_, R_3_, R_4__′_=OH

	Anthocyanidin	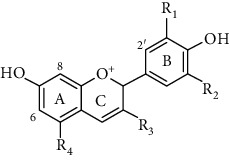	R=H
51	Cyanidin chloride		R_1_=OH
52	Delphinidin chloride		R_2_, R_3_=OH
53	Cyanin chloride		R_1_=OH, R_2_=H, R_3_, R_4_=Oglc
54	Cyanidin-3-*O*-glucoside chloride		R_1_=OH, R_2_=H, R_3_=Oglc
55	Pelargonin chloride		R_1_, R_2_=H, R_3_, R_4_=Oglc
56	Oenin chloride		R_1_, R_2_=OCH_3_, R_3_=Oglc
57	Malvin		R_1_, R_2_=OCH_3_, R_3_, R_4_=Oglc

	Flavans	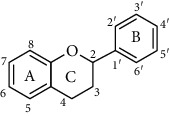	R=H
58	Epicatechin		R_3_, R_5_, R_7_, R_4__′_, R_5__′_=OH
59	Catechin		R_3_, R_5_, R_7_, R_4__′_, R_5__′_=OH
60	Epigallocatechin gallate		R_3_=gallic acid, R_5_, R_7_, R_3__′_, R_4__′_, R_5__′_=OH

Orha: -O-*α*-L-rhamnopyranoside; Oara: -O-*α*-L-arabinofuranoside; Oglc: -O-glucopyranoside; Ogal: -O-*β*-L-galactopyranoside; Cglc: -C-glucopyranoside; Oglcgla: -O-(6-deoxy-*α*-L-mannopyranosyl)-*β*-D-glucopyranoside. The values having no letters in common are significantly different (*P* < 0.05). R is the number in core structure.

## Data Availability

All data generated or analyzed during this study are included in this article.

## Supplementary Materials

Supplementary Materials Table S1: the antioxidant activities of 60 flavonoids determined by DPPH, ORAC, and CAA assays.
