# Supplementary material for: Identification of Six Flavonoids as Novel Cellular Antioxidants and Their Structure-Activity Relationship
Source: Oxid Med Cell Longev. 2020 Sep 19;2020:4150897. doi: 10.1155/2020/4150897 (PMC7525318; doi:10.1155/2020/4150897)
Supplement: Supplementary Materials — Table S1: the antioxidant activities of 60 flavonoids determined by DPPH, ORAC, and CAA assays. [file 4150897.f1.docx]

Table S1 The antioxidant activities of 60 flavonoids determined by DPPH, ORAC and CAA assays

| NO | Flavonoids | DPPH values  (IC_50_,μM) | | ORAC values  (μmol TE/μmol) | | EC50 (μM ) |
| --- | --- | --- | --- | --- | --- | --- |
|  | Flavone | |  | |  |  |
| 1 | Isorhamnetin | | 126.48 ± 4.26^b^ | | 2.63 ± 0.05 | 23.26 ± 1.38^l^ |
| 2 | 7-Methoxy-quercetin | | 34.03 ± 0.61^o^ | | 4.72 ± 0.02 | 10.84 ± 0.34^r^ |
| 3 | Kaempferide | | > 200 | | 1.97 ± 0.02 | >30 |
| 4 | 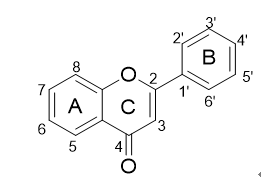Morin | | > 200 | | 5.98 ± 0.01 | 15.23 ± 0.32^q^ |
| 5 | 3-*O*-Methylquercetin | | > 200 | | 4.71 ± 0.12 | 19.53 ± 1.48^n^ |
| 6 | Kaempferol | | > 200 | | 4.17 ± 0.06 | 10.95 ± 0.25^r^ |
| 7 | Quercetin | | 21.52 ± 1.90^q^ | | 5.52 ± 0.04 | 8.77 ± 0.09^s^ |
| 8 | 8-Hydroxy-kaempferol | | > 200 | | 5.05 ± 0.05 | 27.12 ± 2.47^h^ |
| 9  10 | Myricitrin  Quercetin-3-*O*-*α*-arabinofuranose | | 50.87 ± 2.14^k^  71.68 ± 0.06^f^ | | 3.62 ± 0.14  3.34 ± 0.07 | >200  45.12 ± 2.12^d^ |
| 11 | Trifolin | | 45.07 ± 2.12^m^ | | 2.06 ± 0.01 | >200 |
| 12  13  14 | Kaempferol-4'-*O*-glucopyranoside  Kaempferol-7-*O*-glucopyranoside  Kaempferol-3-*O*-arabinoside | | > 200  > 200  > 200 | | 1.73 ± 0.02  3.56 ± 0.04  2.35 ± 0.05 | >200  57.78 ± 3.12^b^  >200 |
| 15 | Isorhamnetin-3-*O*-glucopyranoside | | > 200 | | 1.89 ± 0.02 | >200 |
| 16 | Rutin | | 69.97 ± 1.4^gh^ | | 12.85 ± 0.42 | >200 |
| 17 | Spiraeoside | | > 200 | | 2.12 ± 0.02 | >200 |
| 18 | Myricetin | | 21.26 ± 1.33^q^ | | 6.64 ± 0.03 | 26.73 ± 1.04^i^ |
| 19 | Tangeretin | | > 200 | | 1.02 ± 0.05 | >200 |
| 20 | Chrysin | | > 200 | | 2.34 ± 0.02 | >200 |
| 21 | Baicalein | | > 200 | | 4.11 ± 0.03 | 42.5 ± 0.24^f^ |
| 22 | Apigenin | | > 200 | | 4.27 ± 0.02 | >200 |
| 23  24  25  26  27  28  29 | Luteolin  Cynaroside  Myricetin-3-*O*-galactoside  Quercetin-3-*O*-galactoside  Quercetin-3-*O*-rhamnoside  Quercitrin  Isoquercitrin | | 73.23 ± 0.75^e^  > 200  82.41 ± 2.88^d^  53.34 ± 2.64^j^  47.68 ± 1.60^l^  68.26 ± 1.37^h^  > 200 | | 4.27 ± 0.02  3.58 ± 0.06  3.65 ± 0.03  4.05 ± 0.15  3.97 ± 0.10  3.89 ± 0.12  3.72 ± 0.14 | 18.63 ± 0.61^p^  >200  >200  45.94 ± 0.32^c^  22.79 ± 0.12^m^  >200  >200 |
| 30  31  32 | Vitexin  Orientin  Luteolin6-*C*-glucoside | | > 200  > 200  > 200 | | 6.80 ± 0.42  3.67 ± 0.03  3.45 ± 0.02 | >200  >200  139.21 ± 5.21^a^ |
| 33 | Isovitexin | | > 200 | | 1.62 ± 0.01 | >200 |
| 34 | Galangin | | > 200 | | 2.01 ± 0.06 | 31.23 ± 0.86^g^ |
| 35 | Fisetin | | 25.25 ± 0.62^p^ | | 5.36 ± 0.21 | >200 |
| 36 | Diosmetin | | > 200 | | 5.02 ± 0.14 | >200 |
| 37 | Genkwanin  flavanones | | > 200 | | 1.58 ± 0.03 | >200 |
| 38 | Dihydromyricetin | | > 200 | | 5.25 ± 0.02 | >200 |
| 39 | Taxifolin | | 59.55 ± 3.12^i^ | | 5.26 ± 0.04 | >200 |
| 40 | 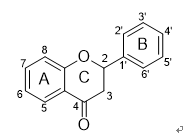Dihydromorin | | > 200 | | 5.29 ± 0.05 | >200 |
| 41 | Neohesperidin | | > 200 | | 3.21 ± 0.12 | >200 |
| 42 | Narirutin | | > 200 | | 3.52 ± 0.04 | >200 |
| 43 | Hesperetin | | > 200 | | 3.62 ± 0.11 | >200 |
| 44 | Hesperidin | | > 200 | | 6.52 ± 0.15 | >200 |
| 45 | Naringenin | | > 200 | | 4.62 ± 0.13 | >200 |
| 46 | Liquiritigenin | | > 200 | | 3.67 ± 0.05 | >200 |
|  | Chalcone | |  | |  |  |
| 47 | 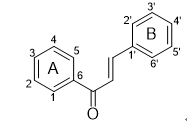Neohesperidin dihydrochalcone | | > 200 | | 4.31 ± 0.01 | >200 |
| 48 | Phloretin | | > 200 | | 3.57 ± 0.02 | 30.96 ± 0.20^g^ |
| 49 | Phlorizin | | > 200 | | 6.43 ± 0.14 | >200 |
| 50 | Isoliquiritigenin | | > 200 | | 3.89 ± 0.04 | >200 |
|  | 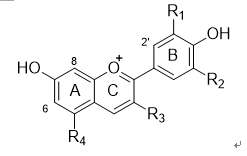Anthocyanidin | |  | |  |  |
| 51 | Cyanidin chloride | | 70.80 ± 2.31^fg^ | | 5.71 ± 0.03 | 19.16 ± 0.16^o^ |
| 52 | Delphinidin chloride | | 36.83 ± 4.26^n^ | | 5.02 ± 0.02 | 24.91 ± 0.12^k^ |
| 53  54 | Cyanin chloride  Cyanidin-3-*O*-glucoside chloride | | > 200  > 200 | | 3.21 ± 0.12  4.63 ± 0.14 | >200  43.75 ± 1.70^e^ |
| 55 | Pelargonin chloride | | > 200 | | 2.74 ± 0.05 | >200 |
| 56 | Oenin chloride | | > 200 | | 4.78 ± 0.15 | >200 |
| 57 | Malvin | | > 200 | | 4.32 ± 0.19 | >200 |
|  | 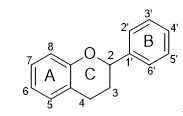Flavans | |  | |  |  |
| 58 | Epicatechin | | 96.031 ± 0.13^c^ | | 4.83 ± 0.13 | >200 |
| 59 | Catechin | | 129.99 ± 5.55^a^ | | 4.93 ± 0.02 | >200 |
| 60 | Epigallocatechin gallate | | 19.13 ± 0.62^r^ | | 6.02 ± 0.14 | 25.91 ± 0.25^j^ |

The values having no letters in common are significantly different (*P* < 0.05).
